# Enhancement of Pb(ii) adsorptive removal by incorporation of UiO-66-COOH into the magnetic graphitic carbon nitride nanosheets†

**DOI:** 10.1039/d4ra00364k

**Published:** 2024-03-18

**Authors:** Sayeh Alvandi, Mojtaba Hosseinifard, Mohsen Bababmoradi

**Affiliations:** a Department of Physics, Iran University of Science and Technology Tehran 16846-13114 Iran babamoradi@iust.ac.ir; b Department of Nano Technology and Advanced Materials, Materials and Energy Research Center Karaj Iran sayeh.alvandi78@gmail.com; c Department of Energy, Materials and Energy Research Center Karaj Iran m.hosseini@merc.ac.ir

## Abstract

Efficient elimination of Lead (Pb(ii)) from aqueous solutions has become a crucial area of focus in the wastewater treatment industry. In this study, novel mesoporous magnetic g-C_3_N_4_/Fe_3_O_4_/UiO-66-COOH was synthesized by combining the acid-functionalized metal–organic framework (MOF) of UiO-66-COOH *via* a facile novel solvothermal method with magnetic graphitic carbon nitride (g-C_3_N_4_/Fe_3_O_4_) sheets to enhance Pb(ii) adsorption in water. The study investigated various influential adsorption parameters, including pH, dosage, contact time, ion concentration, and temperature. The Langmuir model, which depicts monolayer adsorption on a uniform surface, was a more suitable fit for the adsorption isotherms. The kinetics conformed to the pseudo-second-order model, indicating a chemical adsorption mechanism. According to the Langmuir model, the adsorption capacity of g-C_3_N_4_/Fe_3_O_4_/UiO-66-COOH is expected to reach a maximum of 285.8 mg L^−1^. This value is 2.6 times higher than g-C_3_N_4_/Fe_3_O_4_ and 1.6 times higher than UiO-66-COOH. The enhanced adsorption capacity of g-C_3_N_4_/Fe_3_O_4_/UiO-66-COOH is attributed to its superior characteristics, such as abundant functional groups and high surface area which is 2.16 times higher than g-C_3_N_4_/Fe_3_O_4_. The adsorption thermodynamics indicated that the adsorption occurred spontaneously and was characterized as exothermic. g-C_3_N_4_/Fe_3_O_4_/UiO-66-COOH material exhibited good recyclability for up to five runs.

## Abstract 2

Efficient elimination of Lead (Pb(ii)) from aqueous solutions has become a crucial area of focus in the wastewater treatment industry.

## Graphical abstract

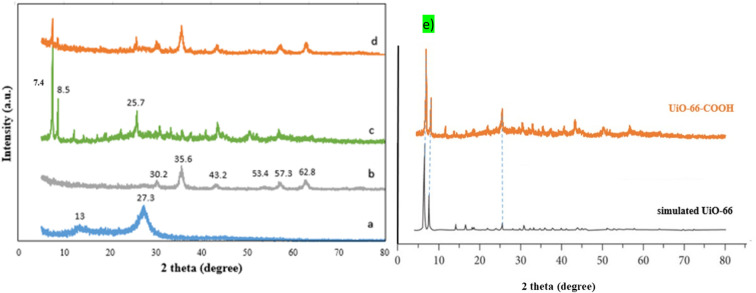

## 1. Introduction

In recent years, the alarming discharge of sewage has led to water contamination by numerous organic and inorganic materials. Among these pollutants, heavy metals have raised alarm, given their characteristics of solubility, oxidation–reduction tendencies, and complex formation. Heavy metals can be a severe threat to human health and the environment, even in low concentrations. Unlike organic pollutants, heavy metals are not easily treatable by biological, physical, or chemical methods.^1^ As such, it is imperative to devise an efficient and effective strategy for removing these pollutants from contaminated water sources. Pb(ii) is a highly toxic and hazardous heavy metal found in wastewater discharged mostly from industries such as electroplating, electrical, steel, and explosive manufacturers. Pb(ii) can cause severe harm to the circulatory system, sense organs, and nervous system in humans and also lead to infant brain damage, loss of voluntary muscle function, and cancer.^2,3^ There are several methods that are used for heavy metals removal such as ion exchange, coagulation, membrane, filtrations, chemical oxidation, catalyst, and adsorption. Among these techniques, adsorption is a highly promising technique that offers numerous advantages, including cost-effectiveness, lack of byproducts, and minimal sludge problems.

One of the appealing adsorbents is graphitic carbon nitride, which in recent years has gained a lot of attention in many fields such as catalysts, photocatalysts, sensors, and photocathodes^4^ due to its low price, easy synthesis methods, and excellent chemical and thermal stability.^5,6^ g-C_3_N_4_ contains functional groups of =N–/–NH–/–NH_2_ forming tri-*s*-triazine rings, establishing many potential adsorption sites.^7^ The lone pair electrons of nitrogen could form electrostatic interaction with cations due to their negative charges, and heavy metal cations could coordinate with these lone pair electrons located between C and N atoms.^8^ The π–π-conjugate interaction and formation of covalent bonds between N functional groups could also result in the adsorptive removal of heavy metal ions.^9^

To easily separate the adsorbent from the solvent, magnetizing the adsorbent could be beneficial.

Fe_3_O_4_, as a supermagnetic nanoparticle, has been combined with many materials due to its facile synthesis methods and low cost.^10^ Fe_3_O_4_ is also a nano-porous material that can adsorb Pb(ii) ions in a short time.^11^ Compositing magnetic Fe_3_O_4_ nanoparticles to GO and g-C_3_N_4_ is an efficient method to raise the adsorption capacity, prevent Fe_3_O_4_ nano materials accumulation, and ease the adsorbent extraction.^12,13^

In recent years, the utilization of porous materials such as activated carbon, zeolites, and MOFs has rapidly increased in the adsorption field since their higher surface area and porosity are beneficial for adsorption.^14^

Metal–organic frameworks offer a fascinating solution to porous crystalline materials. These frameworks are composed of inorganic metal nodes connected by organic ligands. By harnessing the unique features such as large surface area and porosity, and adjustable structures, numerous fields, including sensors, gas sorption, catalysis, and absorption, have shown great interest in these materials.^15^ However, many MOFs, such as MIL-68(Al), Cu-BTC, and Mg-MOF-74 are unstable under acid/alkaline environments and high temperatures.^16,17^

Among MOFs, UiO-66 is structurally stable in water and has resistance to acid and alkali,^18,19^ owing to its phenomenal structure, which consists of Zr_6_O_4_(OH)_4_(CO_2_)_12_ metal nodes linked by twelve traphilic acid (H_2_BDC) ligands.^20^ However, the pristine UiO-66 is unable to adsorb Pb(ii) and needs further modifications.^21^ Therefore, adding functional groups to MOF ligands may be essential to enhance MOF's characteristics. For example, introducing functional groups such as (NH_2_)_2_ and (SH)_2_ to UiO66s ligands can increase adsorption capacity by enhancing Pb(ii) removal through covalent bonding, and chelation.^22^

Also, acidic groups containing sulfur (SH) and carboxyl (COOH) functional groups have been found to possess a strong attraction towards Pb(ii) ions. This phenomenon is due to the ability of these functional groups to donate electrons, which in turn can form coordinate bonds with the positively charged Pb(ii) ions, leading to efficient adsorption of lead.^23^

This study designed a novel separatable adsorbent by synthesizing UiO-66-COOH using a solvothermal process and applying it to a magnetic graphitic carbon nitride substrate *via* a novel facile solvothermal method, which improved the surface area compared to g-C_3_N_4_/Fe_3_O_4_ and enhanced the number of adsorption active sites for Pb(ii) adsorption from water. Batch adsorption experiments were conducted to investigate the effects of dosage, PH, contact time, initial concentration, and temperature on Pb(ii) adsorption. In addition, the study included kinetics, isotherms, thermodynamics, and reusability.

## 2. Experimental

### 2.1. Materials and methods

Lead nitrate (Pb(NO_3_)_2_), urea, FeCl_3_·6H_2_O, FeCl_2_·4H_2_O, ethanol (99%), 1,2.4 benzene tricarboxylic acid (1,2,4 BTC), ZrCl_4_, acetic acid (37%), *n*-hexane, toluene, HCl (37%) and NaOH (99%) were obtained from E. Merc. All the Pb(ii) solutions were prepared with deionized water.

### 2.2. Synthesis of g-C_3_N_4_/Fe_3_O_4_

Urea was heated to 450 °C for 4 : 30 hours in a muffle furnace to pyrolysis into g-C_3_N_4_. Then, the resultant yellow powder was gathered in a desiccator so as not to be exposed to moisture.

To prepare g-C_3_N_4_/Fe_3_O_4_, 0.5 g of g-C_3_N_4_ was ultrasonicated in 50 mL of deionized water for 20 minutes. Next, 1.37 g of FeCl_3_·6H_2_O and 0.50 g of FeCl_2_·4H_2_O were added to the suspension. The mixture was transferred into a two-necked-round-bottomed flask that was attached to a reflux condenser tube and stirred for an hour in an Ar environment. The mixture was then injected with 10 mL of ammonia dropwise. After stirring for another hour at 80 °C, the dark brown precipitates were separated by a magnet, rinsed several times with ethanol and deionized water, and dried at 55 °C for 12 hours.

### 2.3. Synthesis of UiO-66-COOH

The synthesis of UiO-66-(COOH) was conducted following a previously reported procedure with certain modifications.^24^ 0.81 g of ZrCl_4_ and 0.69 g of 1,2,4 BTC were dispersed in 33.3 mL of acetic acid and deionized water (2 : 3) solution. The solution was then transferred to a round-bottom flask with a reflux condenser and stirred with a magnetic stirrer. Then, the mixture was refluxed under an Ar atmosphere for 24 h at 100 °C. After centrifuging, the white precipitates were dried at 55 °C for 8 hours. Then, the powder was soaked in *n*-hexane solution in centrifuge tubes for 3 days, and fresh *n*-hexane was added to the solution each day. The resultant white powder was centrifuged and washed with *n*-hexane. Eventually, the resultant white powder was dried at 80 °C in an oven for 24 hours.

### 2.4. Synthesis of g-C_3_N_4_/Fe_3_O_4_/UiO-66-COOH

**Scheme 1:**
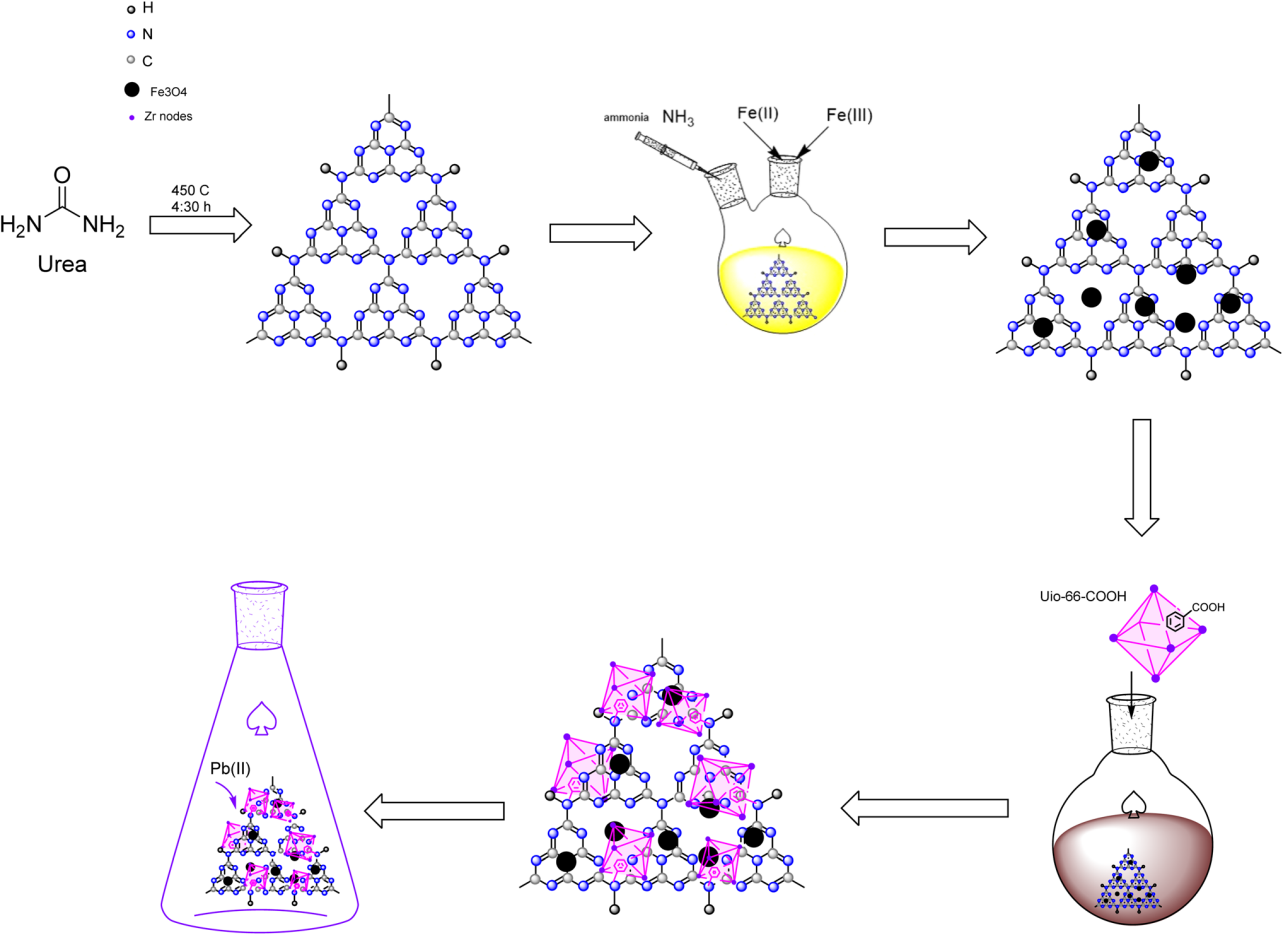
Diagram of the synthesis of g-C_3_N_4_/Fe_3_O_4_/UiO-66-COOH nanocomposite.

0.45 g of magnetic g-C_3_N_4_ was dispersed in 60 mL toluene and stirred in a round bottom flask at room temperature for 5 minutes. The solution was then treated with 0.28 g of UiO-66-COOH. The mixture underwent reflux under an argon environment at a temperature of 85 °C for 24 hours. The resultant product was washed multiple times with ethanol, separated using a magnet, and dried at 55 °C for 12 hours. All the aforementioned synthesis routes are depicted in Scheme 1.

### 2.5. Characterization of the product

Fourier transform infrared (FTIR) spectra were acquired using a DIGILAB FTS 7000 spectrometer with a resolution of 4 cm^−1^ in the range 4000–400 cm^−1^ using the KBr pellet method. TGA (BAHR STA 503 instrument) was performed in an Ar environment heated at a flow rate of 10 mL min^−1^ between 20 °C and 800 °C. The X-ray powder diffraction (XRD) data was obtained using a PHILIPS PW 3710 X-ray diffractometer with Cu and Ka radiation (40 kV, 25 mA) in the 5*θ* to 80*θ* range. Field-emission scanning electron microscopy (FESEM, MIRA3 TESCAN) was used to examine the crystallite size and shape of synthesized materials. The textural parameters were determined by a N_2_ adsorption analysis on a BELSORP-mini II at −196 °C, and the pore size distribution and the surface area were measured by the Barrett–Johner–Halenda (BJH) and Brunauer–Emmett–Teller (BET) methods. The magnetic characteristics were examined by a vibrating sample magnetometer (VSM, Kashan University). Atomic adsorption spectroscopy (AAS, GBC 932 plus) was used to evaluate the concentration of Pb(ii) ions in water.

### 2.6. Adsorption experiments

A stock solution of Pb(ii) was prepared with a concentration of 500 ppm. This was achieved by dissolving 450 mL of Pb(NO_3_)_2_ powder in 500 mL of deionized water. To obtain lead solutions with desired concentrations, the prepared stock solution was diluted. All adsorption experiments were executed by adding an amount of adsorbent to 20 mL of Pb(ii) solutions in 50 mL Erlenmeyers, shaking at 160 rpm in a thermostated shaker at 308 K.

After a certain contact time, the adsorbent was separated by a magnet from the solution, and the remaining lead was estimated by AAS.

The adsorption capacity and efficiency of the adsorbent were determined using eqn (1) and (2), respectively.1
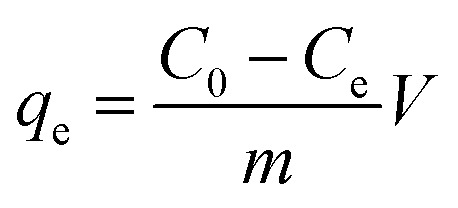
2
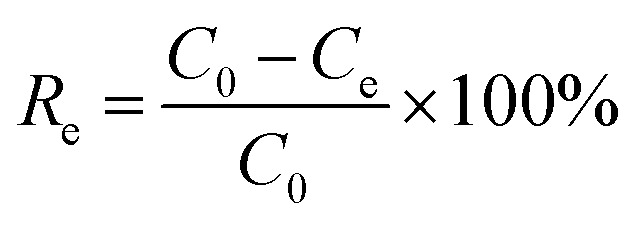


The parameters used for measuring the adsorption of Pb(ii) are *q*_e_ (mg g^−1^) and *R*_e_ (%), which represent the adsorption capacity and the adsorption efficiency, respectively. *V* (L) stands for the volume of the aqueous solution, while *m* (g) represents the mass of adsorbents. Additionally, the initial concentration is denoted by *C*_0_ (mg L^−1^), and the equilibrium concentration is represented by *C*_e_ (mg L^−1^).

To investigate the influence of pH, a 10 ppm Pb(ii) solution was adjusted to pH values between 2 to 7 by 0.1 M HCl and NaOH. Additionally, the effect of adsorbent dosage was also studied by adding 0.25 g L^−1^ to 1 g L^−1^ of adsorbent to 100 ppm Pb(ii) solutions and shaking for 3 hours. Further experiments were conducted at the optimal pH of 6 and dosage of 0.5 g L^−1^.

The adsorption isotherms were studied by adding 0.5 g L^−1^ of adsorbent to solutions, with initial concentrations ranging from 30 ppm to 350 ppm at pH of 6, and allowing them to come into contact for 20 hours. Kinetic studies were carried out by shaking 100 ppm lead solutions for predefined durations ranging from 5 to 180 minutes. To examine the adsorption thermodynamics, several temperatures (298 K, 308 K, 318 K) were used.

## 3. Results and discussion

### 3.1. Characterization

**Fig. 1:**
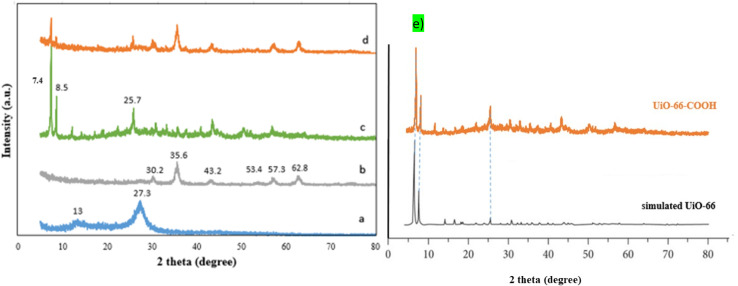
The XRD patterns of (a) g-C_3_N_4_, (b) g-C_3_N_4_/Fe_3_O_4_, (c) UiO-66-COOH, (d) g-C_3_N_4_/Fe_3_O_4_/UiO-66-COOH, (e) comparison of UiO-66-COOH with simulated UiO-66.

As shown in Fig. 1a–d, the phase structure of samples was examined by X-ray diffraction patterns (XRD). The g-C_3_N_4_ exhibits a broad peak at 2*θ* = 13° and a solid peak at 2*θ* = 27.3°, proving the presence of the (100) and (002) diffraction planes related to the aromatic system's in-plane stacking and, the tris-*s*-triazine in-plane structural repeat units.^25^ The g-C_3_N_4_/Fe_3_O_4_ nanocomposite has a broad peak at 2*θ* = 27.3°, indicating that the crystal structure of g-C_3_N_4_ is maintained. However, the low intensity of this peak suggests that the introduction of Fe_3_O_4_ particles has limited the stacking of g-C_3_N_4_ in a direction perpendicular to the [002] axis.^26^ The existence of Fe_3_O_4_ nanoparticles is indicated by the peaks at 2*θ* = 30.2°, 35.6°, 43.2°, 53.4°, 57.3°, and 62.8°.^27^ For pristine UiO-66-COOH, the characteristic diffractions at 2*θ* = 7.4°, 2*θ* = 8.5°, and 2*θ* = 25.7° were similar to previous studies, and according to Fig. 1e, the synthesized UiO-66-COOH displays the same peaks as the simulated UiO-66, indicating their similar topology.^28,29^ All the aforementioned peaks are present in g-C_3_N_4_/Fe_3_O_4_/UiO-66-COOH nanocomposite suggesting UiO-66-COOH crystalline preservation in the new nanocomposite and the successful synthesis.

**Fig. 2:**
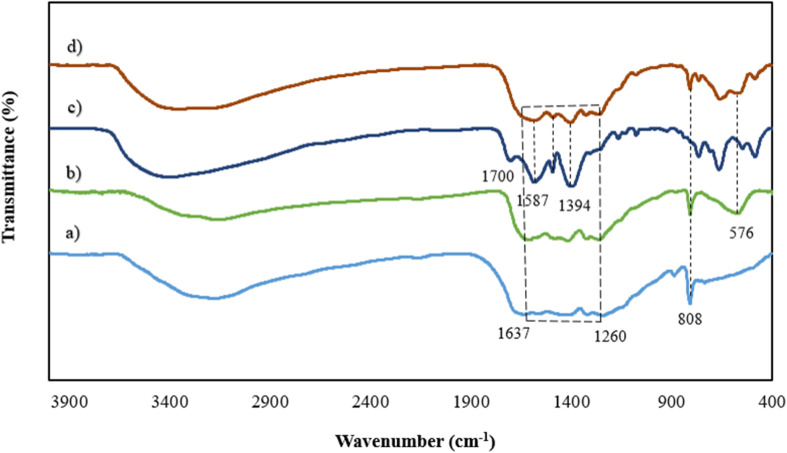
The FTIR spectrums of (a) g-C_3_N_4_, (b) g-C_3_N_4_/Fe_3_O_4_, (c) UiO-66-COOH, (d) g-C_3_N_4_/Fe_3_O_4_/UiO-66-COOH.

As shown in Fig. 2a, the g-C_3_N_4_ FTIR spectrum displays wide peaks of around 3100–3400 cm^−1^ attributed to stretching vibration modes =NH and –NH amines, while the peaks in the area between 1260 and 1637 cm^−1^ are related to the C–N and C=N stretching vibrations. The vibrations of the *s*-triazine ring are associated with the peak at 808 cm^−1^.^25^ The g-C_3_N_4_/Fe_3_O_4_ (Fig. 2b) possesses an additional peak at 576 indicating the presence of the Fe–O bond.^27^ For UiO-66-COOH (Fig. 2c), the existence of free carbonyl groups was verified by the adsorption band at 1700 cm^−1^. The peaks at 1394 and 1587 cm^−1^ are associated with the C=C groups in the benzene ring of H_3_BTC and C–O vibration modes in the carboxyl group. The broadband between 3000 and 3500 is ascribed to OH groups.^29^ However; the peak attributed to carbonyl groups did not appear in the final product (Fig. 2d) suggesting the interaction of carbonyl groups of UiO-66-COOH with g-C_3_N_4_/Fe_3_O_4_. The presence of other peaks mentioned above in the final product implies a successful composition of UiO-66-COOH and g-C_3_N_4_/Fe_3_O_4_.

**Fig. 3:**
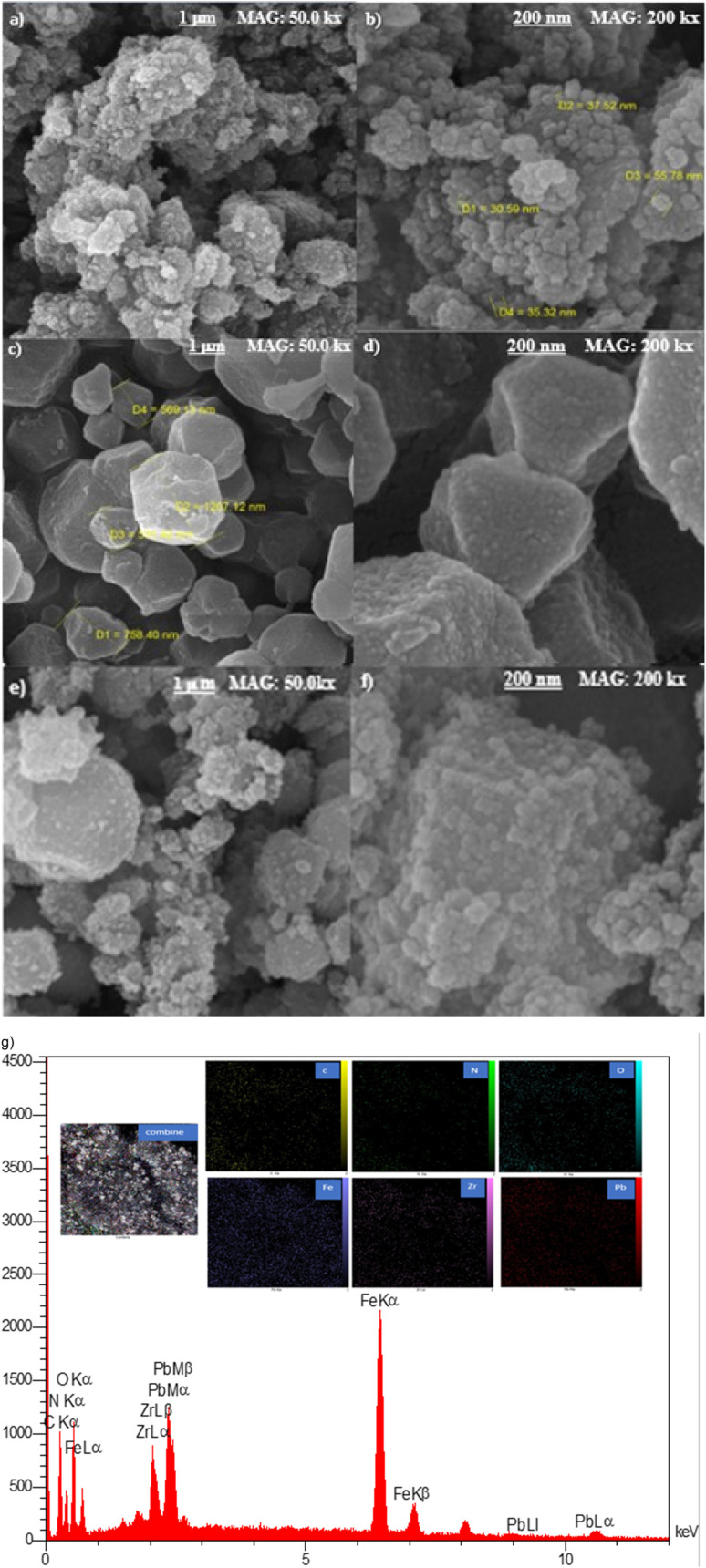
SEM images of (a and b) g-C_3_N_4_/Fe_3_O_4_, (c and d) UiO-66-COOH, (e and f) g-C_3_N_4_/Fe_3_O_4_/UiO-66-COOH and (g) EDX mapping of Pb@g-C_3_N_4_/Fe_3_O_4_/UiO-66-COOH.

The Field-Emission Scanning Electron Microscopy (FESEM) method was used to investigate the morphology of the synthesized materials. As illustrated in Fig. 3a, the surface of g-C_3_N_4_/Fe_3_O_4_ was comprised of spherical nanoparticles that were decorated on plate-like sheets which were consistent with similar studies.^30^ The size of the aforementioned nanospheres ranged from 30 nm to 55 nm, as depicted in Fig. 3b. The UiO-66-COOH exhibits an irregular polyhedral configuration that varies in size from 500 nm to 2 micrometers and features a rough surface with uneven phases along its edges. As shown in Fig. 3e and f, UiO66-COOH particles were successfully incorporated into g-C_3_N_4_/Fe_3_O_4_ nanocomposites, and indicate a close contact between UiO66-COOH and g-C_3_N_4_/Fe_3_O_4_ which according to the FTIR results is due to the interaction between free carbonyl groups of UiO-66-COOH and g-C_3_N_4_/Fe_3_O_4_. The EDX mapping of Pb@g-C_3_N_4_/Fe_3_O_4_/UiO-66-COOH (Fig. 3g), demonstrates the presence of all the elements in a uniform dispersion, and a successful Pb uptake of the surface.

**Fig. 4:**
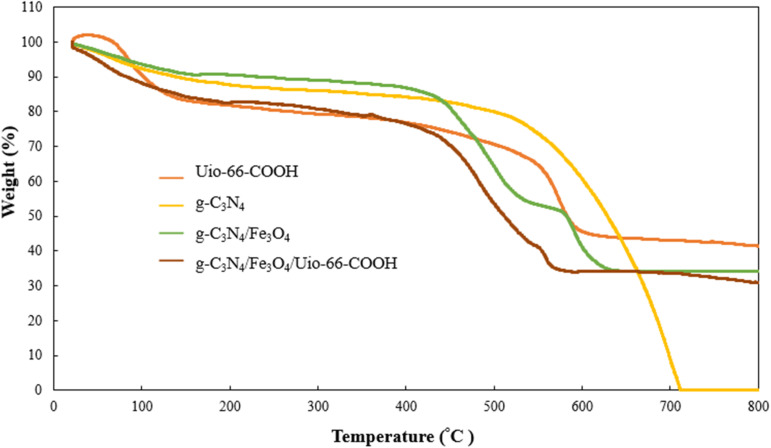
TGA spectra of g-C_3_N_4_, g-C_3_N_4_/Fe_3_O_4_, and g-C_3_N_4_/Fe_3_O_4_/UiO-66-COOH.

To investigate the thermal stability of materials thermogravimetric analysis (TGA) was employed (Fig. 4). The g-C_3_N_4_ exhibited a rapid weight loss at 500 °C to 710 °C within all g-C_3_N_4_ decomposed due to burning.^31^ UiO-66-COOH undergoes two stages of weight loss. The first stage occurs between 20 °C and 350 °C, during which the MOF loses 22% of its weight. This may be attributed to removing the moister adsorbed by MOF and dihydroxylation of free carboxylic groups. The second stage occurs between 350 °C to 600 °C and is characterized by a weight loss of 43% due to the decomposition of the framework.^29^ Both g-C_3_N_4_/Fe_3_O_4_ and g-C_3_N_4_/Fe_3_O_4_/UiO-66-COOH display 65% weight loss in three stages. These nanocomposites have lower decomposition temperatures than pristine g-C_3_N_4_ due to increased active sites for oxygen adsorption.^32^ For g-C_3_N_4_/Fe_3_O_4_, there's an endothermic peak between 530 °C and 600 °C associated with the phase shift from magnetite (Fe_3_O_4_) to hematite (FeO) structure. The FeO phase is thermodynamically stable above 570 °C before declining as a result of the deoxidation of FeO.^33^ The g-C_3_N_4_/Fe_3_O_4_/UiO-66-COOH displays similar behavior to the g-C_3_N_4_/Fe_3_O_4_ since g-C_3_N_4_/Fe_3_O_4_ as a substrate possesses a higher mass ratio. However, the endothermic peak attributed to the Fe_3_O_4_ phase shift is less significant due to the addition of the UiO-66-COOH particles. The g-C_3_N_4_/Fe_3_O_4_/UiO-66-COOH loses 20% of its weight due to the removal of moister, oxidation, and dihydroxylation of free carboxylic groups of MOFs, between 20 °C and 400 °C. Also, 57% weight loss occurs between 400 °C and ∼600 °C due to the decomposition of MOF.

**Fig. 5:**
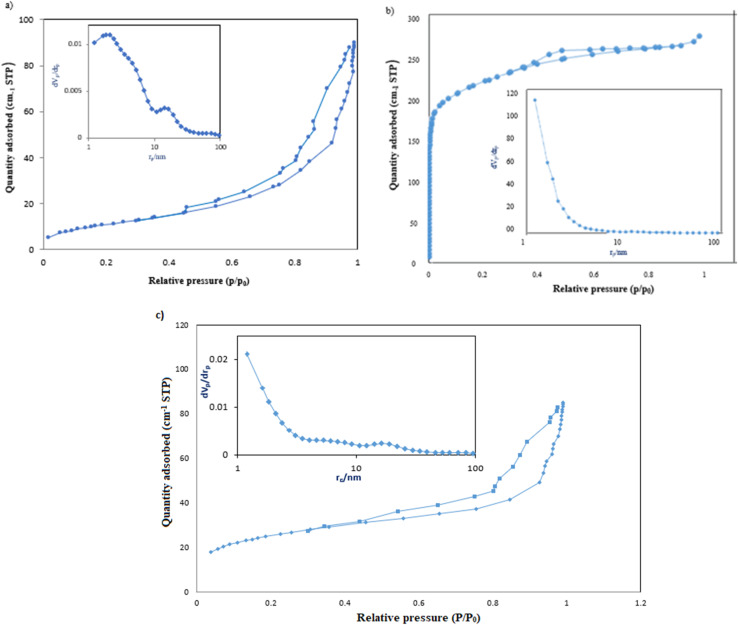
The adsorption and desorption, and BJH plots of (a) g-C_3_N_4_/Fe_3_O_4_, (b) UiO-66-COOH, (c) g-C_3_N_4_/Fe_3_O_4_/UiO-66-COOH.

The adsorption–desorption isotherms of g-C_3_N_4_/Fe_3_O_4_, UiO-66-COOH, and g-C_3_N_4_/Fe_3_O_4_/UiO-66-COOH were analyzed using the Brunauer–Emmett–Teller (BET) technique, and the pore structure of synthesized materials was determined using Barrett–Johner–Halenda (BJH) (Fig. 5a–c). UiO-66-COOH exhibited a type I isotherm, indicating the presence of micropores.^28^ The specific surface area of UiO-66-COOH was determined to be 540 m^2^ g^−1^, and the mean pore size was found to be 1.10 nm through BJH analysis. Conversely, both g-C_3_N_4_/Fe_3_O_4_ and g-C_3_N_4_/Fe_3_O_4_/UiO-66-COOH demonstrated a type IV isotherm, indicative of a mesoporous structure.^34^ The specific surface area of g-C_3_N_4_/Fe_3_O_4_/UiO-66-COOH was calculated to be 89.82 m^2^ g^−1^, which is 2.16 times that of g-C_3_N_4_/Fe_3_O_4_ surface area (41.54 m^2^ g^−1^) due to the addition of UiO-66-COOH particles. However, the pore diameter of g-C_3_N_4_/Fe_3_O_4_/UiO-66-COOH was found to be 5.80 nm, which is significantly greater than that of UiO-66-COOH. Nonetheless, the incorporation of UiO-66-COOH into g-C_3_N_4_/Fe_3_O_4_ led to a reduction in pore diameter from 13.36 nm to 5.89 nm. The high specific surface area and pore diameter of g-C_3_N_4_/Fe_3_O_4_/UiO-66-COOH render it advantageous for adsorption applications (Table 1).

**Table 1:** BET parameters of samples

Sample	*a*_s_ (m^2^ g^−1^)	Mean pole diameter (nm)
g-C_3_N_4_/Fe_3_O_4_	41.54	13.56
UiO-66-COOH	580	2.1
g-C_3_N_4_/Fe_3_O_4_/UiO-66-COOH	89.82	5.80

**Fig. 6:**
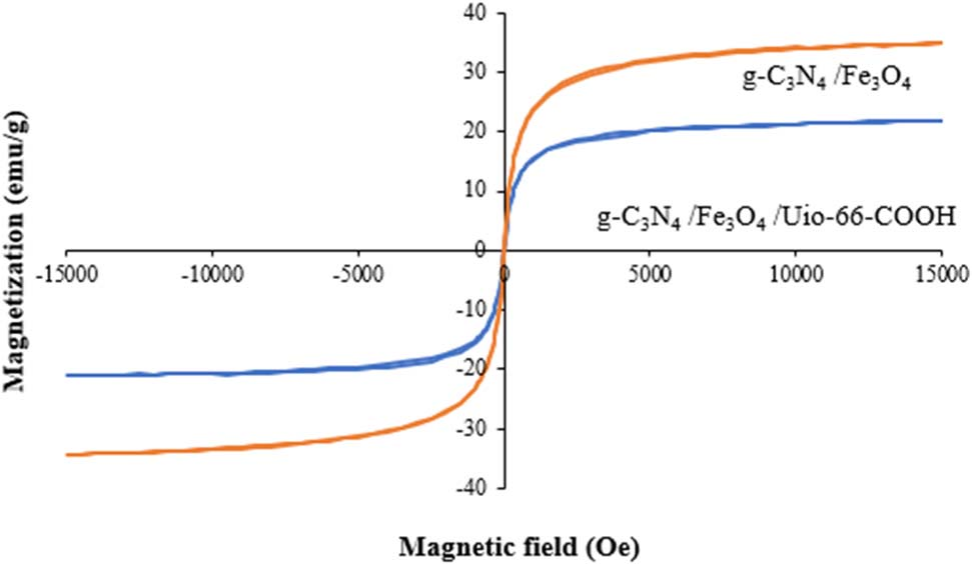
VSM of g-C_3_N_4_/Fe_3_O_4_ and g-C_3_N_4_/Fe_3_O_4_/UiO-66-COOH.

A vibrating sample magnetometer (VSM) was employed at room temperature to evaluate the magnetic characteristics of the samples in the field range of −15 to +15 kOe. As shown in Fig. 6, the magnetic saturation strengths (*M*_S_) of g-C_3_N_4_/Fe_3_O_4_ and g-C_3_N_4_/Fe_3_O_4_/UiO-66-COOH are 34.7 and 20.7 emu g^−1^. The lower amount of *M*_s_ of g-C_3_N_4_/Fe_3_O_4_/UiO-66-COOH is attributed to adding non-magnetic UiO-66-COOH particles to the nanocomposite. However, the hysteresis loops of both samples displayed zero remanence and coercivity, implying supermagnetic properties, suitable for the magnetic separation of samples from water.

### 3.2. Batch adsorption experiments

**Fig. 7:**
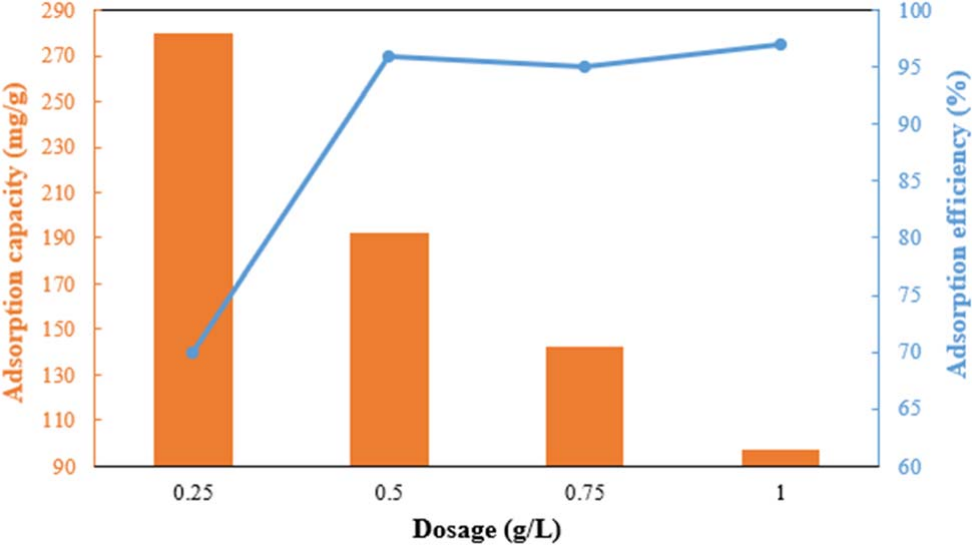
Effect of g-C_3_N_4_/Fe_3_O_4_/UiO-66-COOH dosage on Pb(ii) adsorption capacity and efficiency.

The dosage of the adsorbent has a major influence on adsorption efficiency (Fig. 7). The adsorption efficiency rapidly increases from 70% to 96% when the dose is raised from 0.25 to 0.5 g L^−1^ due to the increase in active sites. However, the efficiency slightly decreases between 0.5 and 0.75 g L^−1^ due to the aggregation of adsorbent particles and overlapping of adsorption sites.^35,36^ The adsorbent's capacity decreases significantly from 280 mg g^−1^ to 97 mg g^−1^ in the 0.25 g L^−1^ to 1 g L^−1^ range. Therefore, a dosage of 0.5 g L^−1^ of g-C_3_N_4_/Fe_3_O_4_/UiO-66-COOH was chosen based on its excellent efficiency and capacity.

**Fig. 8:**
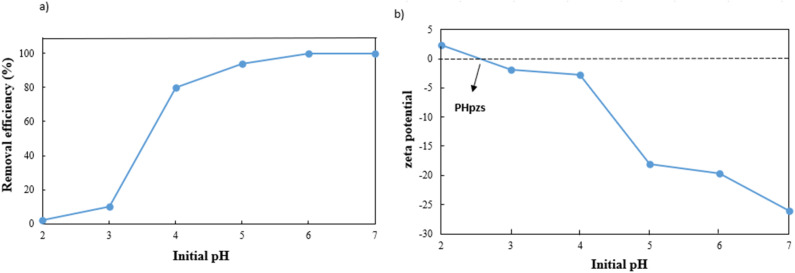
(a) Effect of pH on Pb(ii) adsorption efficiency, and (b) zeta potential of g-C_3_N_4_/Fe_3_O_4_/UiO-66-COOH.

The pH is an influential aspect in the adsorption process since it determines the adsorbate speciation and adsorbent surface charge. Lead is mainly present as Pb^2+^ when the pH is less than 5.8. When the pH value is between 5.8 and 7, both Pb^2+^ and Pb(OH)^+^ forms are prevalent. But as the pH value rises beyond 7, lead can react with the additional hydroxide to create Pb(ii)(OH)_2_ flocculated precipitates, which cannot be absorbed by an adsorbent.^37^ Therefore, the pHs between 2 and 7 were investigated in this study, where 0.5 g L^−1^ of g-C_3_N_4_/Fe_3_O_4_/UiO-66-COOH was added to Pb(ii) solutions (10 ppm), shaking for 20 hours. As shown in Fig. 8a, the adsorption efficiency at pH of 2 is low, which could be attributed to the existence of hydrogen ions (H^+^) occupying adsorption sites. According to zeta potential studies, the surface of the g-C_3_N_4_/Fe_3_O_4_/UiO-66-COOH has a positive charge at pH of 2, causing electrostatic repulsion between the g-C_3_N_4_/Fe_3_O_4_/UiO-66-COOH surface and Pb^+^ cations. As shown in Fig. 8b, the point of zero charge (pH_pzs_) of g-C_3_N_4_/Fe_3_O_4_/UiO-66-COOH is 2.5, indicating that the g-C_3_N_4_/Fe_3_O_4_/UiO-66-COOH surface is charged negatively in pH environments higher than 2.5. An increase in pH levels causes a reduction in the concentration of hydrogen ions (H^+^), causing the adsorbent's surface charge to become less positive. This causes an increase in the number of vacant active sites as well as an increase in the electrostatic attraction force between the Pb(ii) cations and the negatively charged adsorption surface. Consequently, there is a marked enhancement in the removal efficiency. Remarkably, the adsorption efficiency of this particular experiment reached the maximum level of 100% at pH of 6 and 7. Therefore, all subsequent experiments were carried out at these pH values to obtain the maximum adsorption possible.

**Fig. 9:**
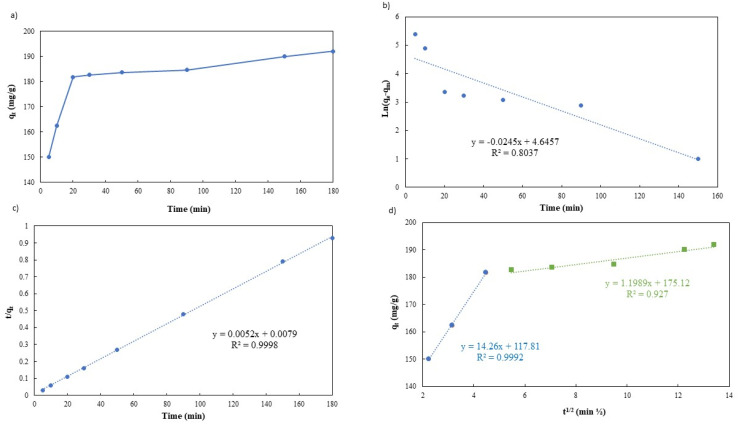
(a) Effect of Pb(ii) adsorption time on g-C_3_N_4_/Fe_3_O_4_/UiO-66-COOH, (b) pseudo-first-order linear plot, (c) pseudo-second-order linear plot, and (d) intra-particle diffusion model.

In order to fully comprehend the process of adsorption, it is essential to study its kinetics. As illustrated in Fig. 9a, the adsorption capacity rapidly increases within the first 20 minutes of the process, owing to the abundance of unoccupied sites on the g-C_3_N_4_/Fe_3_O_4_/UiO-66-COOH surface and a high concentration gradient of Pb(ii) in the solution. It is noteworthy that during this time interval, 90% of Pb(ii) removal occurs. Subsequently, the adsorption capacity gradually increases over time, eventually reaching a state of equilibrium capacity of 192 mg g^−1^ after 180 minutes. This limited rate could be attributed to the active sites being filled by Pb(ii) ions.

The adsorption kinetic data were characterized by the pseudo-first-order model, the pseudo-second-order model, and the Weber–Morris intraparticle diffusion model, the linear versions of each model could be illustrated by eqn (3)–(5) respectively:

3 ln(*q*_e_ − *q*_*t*_) = ln(*q*_e_) − *K*_1_*t*

4
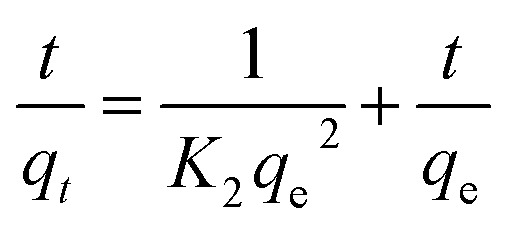

5 *q*_*t*_ = *K*_i_·*t*^1/2^ + *C*_i_

where the variables *K*_1_ (L min^−1^), *K*_2_ (g mg^−1^ min^−1^), and *K*_i_ (mg g^−1^ min^−1/2^) signify the rate constant of the pseudo-first model, pseudo-second model, and intraparticle diffusion rate constant respectively. The adsorption capacities at equilibrium and contact time are represented by *q*_e_ (mg g^−1^) and *q*_*t*_ (mg g^−1^), respectively, and *C*_i_ is the constant associated with the boundary layer thickness.

Table 2 shows the kinetic parameters for each model, and Fig. 9b and c display the pseudo-first-order model and pseudo-second-order linear graphs. With a remarkable correlation coefficient of 0.9988, the pseudo-second-order model exhibits the greatest match, inferring that chemical adsorption was the main mechanism involved in the process.^38,39^ The equilibrium predicted by this model is in good agreement with experimental data.

**Table 2:** Kinetic parameters of Pb(ii) adsorption onto g-C_3_N_4_/Fe_3_O_4_/UiO-66-COOH

Kinetic models	*R* ^2^	*K*	*q*_e_ (mg g^−1^)
Pseudo-first-order	0.8037	0.0245 (L min^−1^)	25.03
Pseudo-second-order	0.9998	0.0034 (g mg^−1^ min^−1^)	192.30
Intra-particle diffuse	*R*_1_ = 0.9992	*K*_1_ = 14.26 (mg g^−1^ min^−1/2^)	*C*_1_ = 117.8
	*R*_2_ = 0.9270	*K*_2_ = 1.989 (mg g^−1^ min^−1/2^)	*C*_2_ = 175.12

Fig. 9d presents the Weber–Morris intraparticle diffusion model plot which could not be fitted by a single straight line but could be divided into two linear sections suggesting that there are other rate-limiting steps besides intraparticle diffusion.^40^ The first step (the initial 20 minutes) suggests a rapid diffusion of Pb(ii) particles from the solution to the external surface of g-C_3_N_4_/Fe_3_O_4_/UiO-66-COOH or boundary layer. The process of intraparticle diffusion, which occurs during the second stage of adsorption, is responsible for the migration of Pb(ii) from the exterior surface to the internal pores of g-C_3_N_4_/Fe_3_O_4_/UiO-66-COOH. A lower *K* value indicates a lower diffusion rate.

**Fig. 10:**
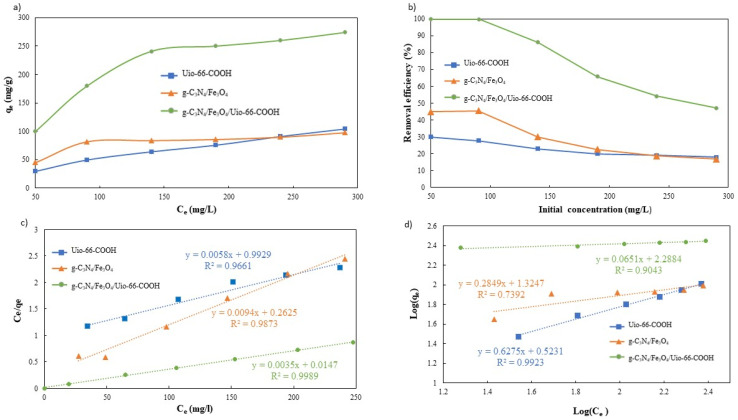
(a) Effect of the initial Pb(ii) concentration on the adsorption capacity, (b) effect of the initial Pb(ii) concentration on the adsorption efficiency, (c) the Langmuir linear plot, and (d) the Freundlich linear plot.

To validate the superiority of the modified g-C_3_N_4_/Fe_3_O_4_/UiO-66-COOH over pristine UiO-66-COOH and g-C_3_N_4_/Fe_3_O_4_, it is essential to study the influence of Pb(ii) concentration and adsorption isotherms. Therefore, for each adsorbent, the initial concentrations ranging from 50 to 290 mg L^−1^ were examined. As Pb(ii) initial concentration increases, the adsorption capacity increases, as the net adsorbed amount of Pb(ii) increases (Fig. 10a). However, the adsorption efficiency decreases due to the adsorbent's active sites being occupied (Fig. 10b). The capacity gradually attained saturation as the concentration of lead escalated. The g-C_3_N_4_/Fe_3_O_4_/UiO-66-COOH exhibits a better performance as it yielded higher capacities and efficiencies since it contains a higher surface area compared to g-C_3_N_4_/Fe_3_O_4_ and a greater number of functional groups as adsorption active sites compared to UiO-66-COOH.

The collected data was analyzed using Freundlich and Langmuir isotherm models to predict the behavior of adsorptive removal and determine the maximum adsorbent's adsorption capacity. The linear versions of the Langmuir and Freundlich models were illustrated using the eqn (6) and (7), respectively:6
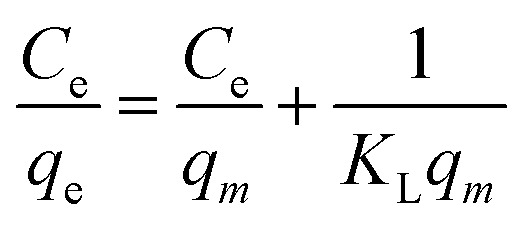
7
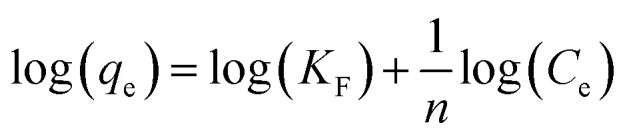
where *C*_e_ (mg L^−1^) and *q*_e_ (mg g^−1^) represent the equilibrium concentration and adsorption capacity of Pb(ii) ions, respectively, the maximum adsorption capacities of the adsorbents are indicated by the symbol *q*_max_ (mg g^−1^). The Langmuir and Freundlich equilibrium constants are represented as *K*_L_ (L mg^−1^) and *K*_F_ (L g^−1^), respectively, and *n* stands for the dimensionless coefficient of the Freundlich model.

The isotherms characteristic parameters for Langmuir and Freundlich models are shown in Table 3 and the corresponding linear graphs are depicted in Fig. 10c and d. The Langmuir model demonstrated higher correlation coefficients concerning the Freundlich model for both the g-C_3_N_4_/Fe_3_O_4_/UiO-66-COOH and g-C_3_N_4_/Fe_3_O_4_, indicating monolayer adsorption on a homogenous surface.^41^ The predicted maximum capacity of g-C_3_N_4_/Fe_3_O_4_/UiO-66-COOH by the Langmuir model is 285.7 mg g^−1^, while g-C_3_N_4_/Fe_3_O_4_ and UiO-66-COOH yielded much lower maximum capacities of 106.38 and 172.4 mg g^−1^. The Freundlich model contains a higher correlation coefficient for pristine UiO-66-COOH suggesting a multilayer heterogamous adsorption for UiO-66-COOH.^42^

**Table 3:** Comparison of g-C_3_N_4_/Fe_3_O_4_/UiO-66-COOH with other studies

Adsorbents	*q*_max_ (mg g^−1^)	pH	Temperature (K)	References
g-C_3_N_4_	65.6	5	298	43
S_3.9%_-g-C_3_N_4_	52.63	4.5	318	44
MNPs-NH_2_	40.10	5	298	45
rGO-Fe_3_O_4_	30.68	7	298	46
UiO-66-NH_2_	166.74	6	303	47
UiO-66-PTC	200.2	5	303	37
g-C_3_N_4_/MnO_2_	204.1	4–7	318	40
g-C_3_N_4_/Fe_3_O_4_/UiO-66-COOH	285.7	6–7	308	This work

As shown in Table 3, g-C_3_N_4_/Fe_3_O_4_/UiO-66-COOH exhibits superior adsorption capabilities compared to similar materials.

The Langmuir parameter (*R*_L_), which could be calculated using eqn (8), is used to determine if the adsorption process is feasible.8
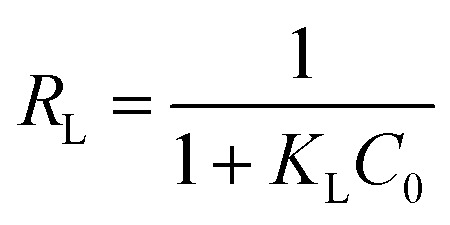
where, *K*_L_ and *C*_0_ denote the Langmuir constant and the initial concentration of Pb(ii), respectively. The adsorption is regarded as favorable when 0 < *R*_L_ < 1, whereas *R*_L_ > 1 implies an unfavorable process.^43^ Since the *R*_L_ calculated for g-C_3_N_4_/Fe_3_O_4_/UiO-66-COOH ranges between 0.014 and 0.080, the adsorption is feasible. The adsorption isotherm parameters for each model are reported in Table 4.

**Table 4:** Absorption isotherm modeling parameters derived from Langmuir model and Freundlich model

Langmuir coefficients					Freundlich coefficients		
Sample name	*Q*_m_ (mg g^−1^)	*K*_L_ (L mg^−1^)	*R* ^2^	*R*_L_	*K*_F_ (L g^−1^)	1/*n*	*R* ^2^
g-C_3_N_4_/Fe_3_O_4_	106.38	0.036	0.9873	0.0877 < *R*_L_ < 0.357	21.12	0.284	0.7304
UiO-66-COOH	172.41	0.006	0.9661	0.365 < *R*_L_ < 0.769	3.33	0.624	0.9907
g-C_3_N_4_/Fe_3_O_4_/UiO-66-COOH	285.71	0.192	0.9989	0.017 < *R*_L_ < 0.094	194.26	0.065	0.9411

To investigate the impact of temperature and study the thermodynamics of adsorption, 200 ppm of lead solutions with 0.5 g L^−1^ dose of adsorbent were shaken for 20 hours at pH of 6 under different temperatures.

**Fig. 11:**
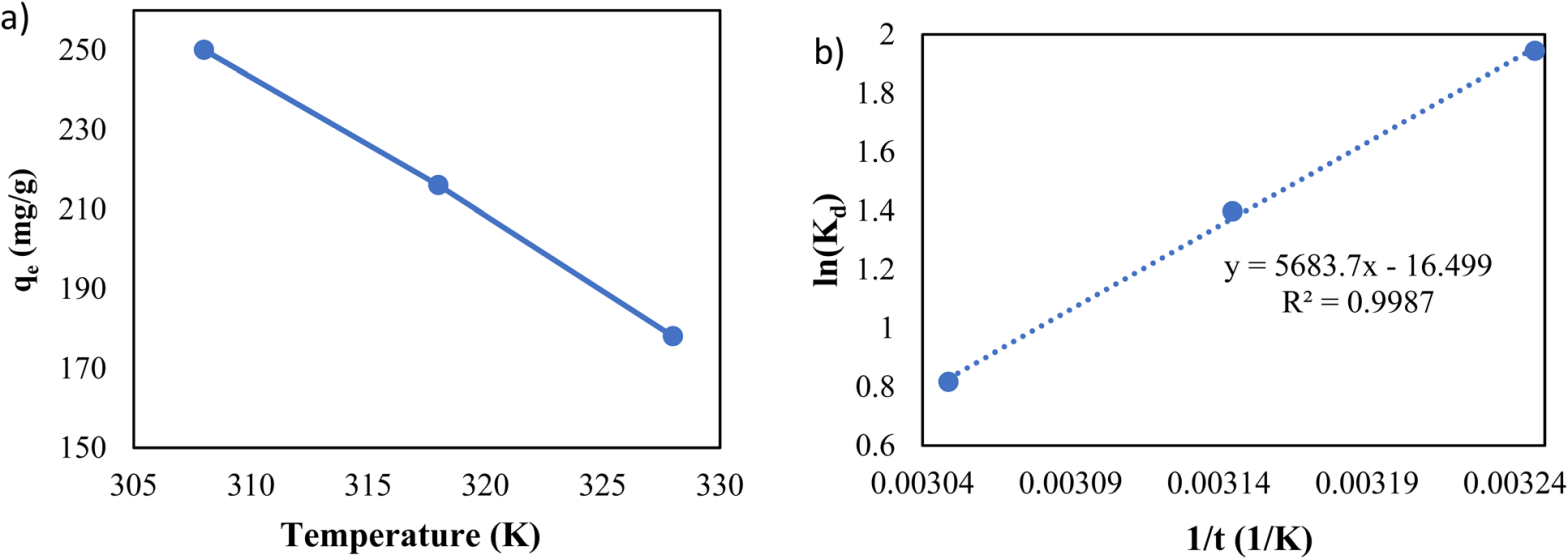
(a) The effect of temperature, and (b) Van't Hoff plot.

As shown in Fig. 11a, the temperature is an influential parameter in the adsorption process. The temperature increase has a negative impact on adsorption capacity, demonstrating that the adsorption of Pb(ii) onto g-C_3_N_4_/Fe_3_O_4_/UiO-66-COOH nanocomposites is an exothermic process.

The thermodynamic parameters of adsorption were calculated as follows:9
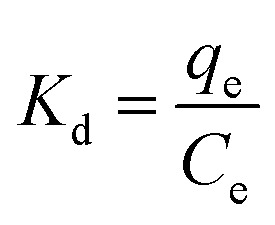

10 Δ*G*^0^ = −*RT* ln(_Kd_)

11
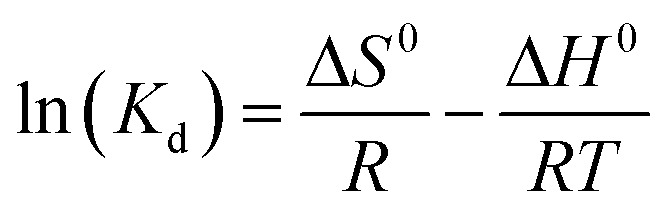
where *K*_d_ (L g^−1^) is the thermodynamic distribution coefficient, calculated by dividing equilibrium capacity by Pb(ii) equilibrium concentration, *T* is the absolute temperature (K), *R* is the universal gas constant (8.314 J mol^−1^ K^−1^), and Δ*H*^0^ and Δ*S*^0^ are enthalpy and entropy.

Eqn (11) states that the slope and intercept of the Van't Hoff plot (Fig. 11b) are equivalent to −Δ*H*^0^/*R* and Δ*S*^0^/*R*. Table 4 shows the calculated thermodynamic parameters.

The Δ*G*^0^ values of all temperatures are negative, implying that Pb(ii) adsorption on g-C_3_N_4_/Fe_3_O_4_/UiO-66-COOH surface is a spontaneous and thermodynamically favored process. However, as the temperature increases, Δ*G*^0^ becomes less negative, implying that adsorption becomes less favorable in high temperatures.^48^

The negative amount of enthalpy (*H*^0^) demonstrates an exothermic adsorption process.^49^ The *S*^0^ parameter was found to be −137.17 J mol^−1^ K^−1^, suggesting that the Pb(ii) was distributed far more chaotically in the aqueous environment than on the surface of the solid phase (adsorbent) (Table 5).^50^

**Table 5:** Thermodynamic parameters for adsorption of Pb(ii) onto g-C_3_N_4_/Fe_3_O_4_/UiO-66-COOH nanocomposites

T(K)	*q*_e_ (mg g^−1^)	*K*_d_	Δ*G*^0^ (kJ mol^−1^)	Δ*H*^0^ (kJ mol^−1^)	Δ*S*^0^ (J mol^−1^ K^−1^)	*R* ^2^
308	250	3.846	−4.976			
318	212	2.634	−3.693	−47.254	−137.17	0.9987
328	178	1.762	−2.229			

**Fig. 12:**
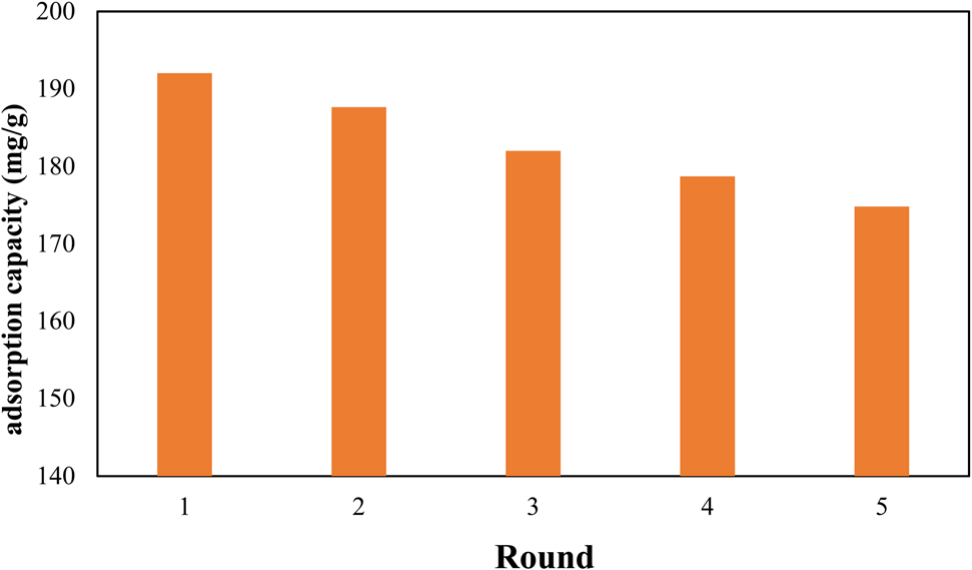
Recycling of g-C_3_N_4_/Fe_3_O_4_/UiO-66-COOH.

Since the reuse and recycling of adsorbent is cost-effective, the recycling of the nanocomposite was also investigated. Therefore, after each adsorption run Pb@g-C_3_N_4_/Fe_3_O_4_/UiO-66-COOH was washed with 0.5 M NaOH solution, and then 50 mg of regenerated nanocomposite was shaken in 100 mL of 100 ppm Pb(ii) solution for 20 h. After 5 cycles, the removal efficiency was reduced to 91.2% of its original capacity, demonstrating good reusability (Fig. 12). The X-ray diffraction (XRD) pattern of the reused g-C_3_N_4_/Fe_3_O_4_/UiO-66-COOH material was analyzed, and compared with that of the fresh sample, as presented in Fig. S1.† The results reveal that no additional peaks were observed in the XRD pattern of the reused material, and no peak was found to disappear, indicating the absence of any structural changes in the material upon usage. The SEM image of reused g-C_3_N_4_/Fe_3_O_4_/UiO-66-COOH (Fig. S2†) also demonstrates the material's integrity remains intact during the reuse studies.

## 4. Conclusions

In this study, a novel mesoporous super-magnetic g-C_3_N_4_/Fe_3_O_4_/UiO-66-COOH nanocomposite was synthesized *via* novel and facile solvothermal methods, and FE-SEM, VSM, TGA, XRD, FT-IR, and BET studies were utilized to identify the nanocomposite. The batch adsorption experiments were executed to evaluate the nanocomposite's ability to adsorb Pb(ii). The nanocomposite reached equilibrium after 180 min with 0.5 g L^−1^ dosage of g-C_3_N_4_/Fe_3_O_4_/UiO-66-COOH at pH of 6 and *T* = 308 K. The g-C_3_N_4_/Fe_3_O_4_ nanosorbent was synthesized through an effective and novel method, however, its capacity was limited despite being a low-cost and easily separable sorbent. The adsorption capacity predicted by the Langmuir method demonstrates that g-C_3_N_4_/Fe_3_O_4_/UiO-66-COOH was found to be 2.60 had 2.60 times higher capacity than g-C_3_N_4_/Fe_3_O_4_ and 1.65 times higher than pristine UiO-66-COOH. This enhancement in performance owes to the material's superior surface area, which is approximately twice that of g-C_3_N_4_/Fe_3_O_4_. Additionally, g-C_3_N_4_/Fe_3_O/UiO-66-COOH contains more functional groups than pristine UiO-66-COOH, further contributing to its increased adsorption capacity. The adsorption isotherms applied to the Langmuir model, suggest that a monolayer adsorption occurred on a uniform layer. The kinetic studies showed that the pseudo-second-order model best fit the data, with chemical adsorption being the main driving force of the process. An increase in pH value from 2 to 7 has been found to improve the adsorption efficiency significantly. This can be attributed to the pivotal role of electromagnetic force in the adsorption process. According to thermodynamic analysis, the process of adsorption is both exothermic and simultaneous. The nanocomposite demonstrated a strong removal rate even after five rounds, highlighting its potential for use in practical applications. Overall, the easy procedure and remarkable adsorptive performance of g-C_3_N_4_/Fe_3_O_4_/UiO-66-COOH make it a promising candidate for the remediation of lead-contaminated water.

## Conflicts of interest

The authors declare no competing interests.

## Supplementary Material

RA-014-D4RA00364K-s001
