# Supplementary material for: Enhancement of Pb(ii) adsorptive removal by incorporation of UiO-66-COOH into the magnetic graphitic carbon nitride nanosheets
Source: RSC Adv. 2024 Mar 18;14(13):8990–9002. doi: 10.1039/d4ra00364k (PMC10945373; doi:10.1039/d4ra00364k)
Supplement: RA-014-D4RA00364K-s001 [file RA-014-D4RA00364K-s001.pdf]

Electronic Supplementary Material (ESI) for RSC Advances.
This journal is © The Royal Society of Chemistry 2024

# Electronic Supporting information

## Enhancement of Pb(II) adsorptive removal by incorporation of UiO-66-COOH into the magnetic graphitic carbon nitride nanosheets

Sayeh Alvandi, [ab] Mojtaba Hosseinifard,[c*] Mohsen Bababmoradi,[a]

[a] *Department of Physics, Iran University of Science and Technology, Tehran 16846-13114, Iran. E-mail:* *babamoradi@iust.ac.ir*

[b] *Department of Nano Technology and Advanced Materials, Materials and Energy Research Center, Karaj, Iran. E-mail:* *sayeh.alvandi78@gmail.com*

[c] *Department of Energy, Materials and Energy Research Center, Karaj, Iran. E-mail:* *m.hosseini@merc.ac.ir*

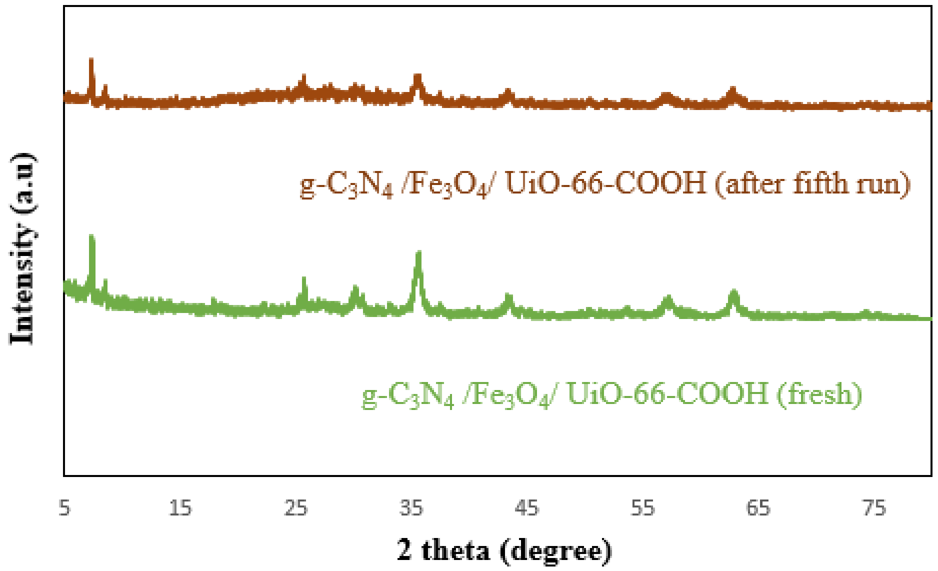


**Fig. S1** XRD patterns of g-$C_3N_4$ /$Fe_3O_4$/ UiO-66-COOH: fresh and after fifth run.

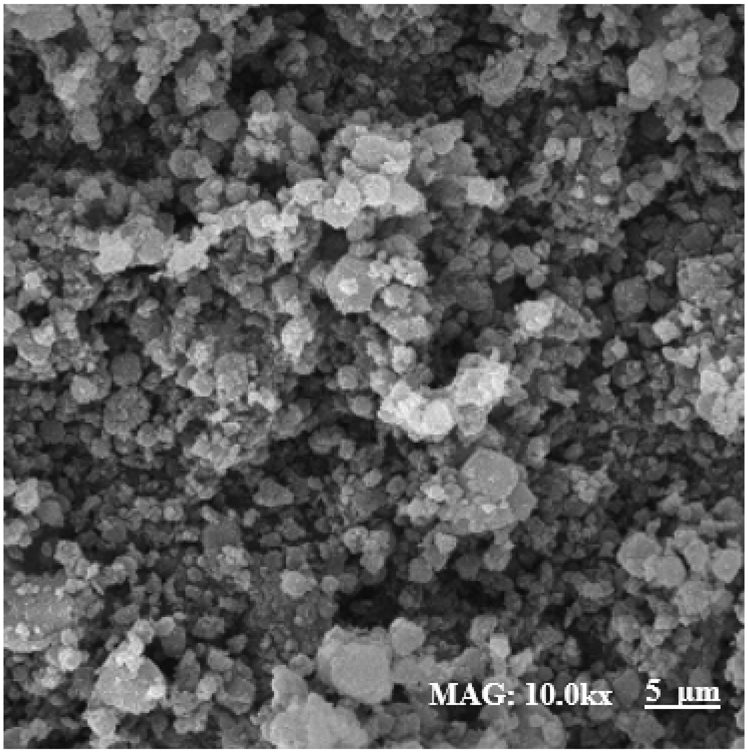


**Fig. S2** SEM image of reused g-$C_3N_4$ /$Fe_3O_4$/ UiO-66-COOH.
